# Portal Vein Thrombosis Due to an Increase in Dose of Testosterone in a Young Man with Klinefelter Syndrome

**DOI:** 10.7759/cureus.1823

**Published:** 2017-11-06

**Authors:** Waseem Amjad, Salma Khatoon, Twara Tarasaria, Gulru Sharifova

**Affiliations:** 1 Forest Hills Hospital, Northshore-Long Island Jewish Health System; 2 Medicine, Northwell-long Island Jewish Forest Hills Hospital

**Keywords:** klinefelter syndrome, thrombosis, klinefelter syndrome, thrombosis, testosterone replacement therapy

## Abstract

Klinefelter syndrome (KS) is associated with increased incidence of thrombotic events. Hypofibrinolysis is associated with increased risk of thromboembolism. Although testosterone replacement therapy (TRT) inhibits the hypofibrinolysis, it can still cause thrombosis paradoxically due to increased dose and duration of use. Herein, we present a case of a young male diagnosed with KS who was taking testosterone. The dose was increased to boost the energy levels, and the patient presented with abdominal pain. Computed tomography (CT) of the abdomen showed extensive portal vein thrombosis. He was started on enoxaparin followed by apixaban. Studies need to be done regarding the need for thromboembolism prophylaxis in patients on TRT.

## Introduction

Klinefelter syndrome (KS), also known as 47XXY, is the most common chromosomal aneuploidy in males and seen in 1/1,000 live male births [1]. Thrombotic events, such as venous thromboembolism (VTE), deep venous thrombosis (DVT), and pulmonary embolism (PE), are five to 20 times more common in men with KS [2]. We present a case of a young male who developed extensive portal vein thrombosis after an increase in the dose of hormonal therapy.

## Case presentation

**Figure 1:**
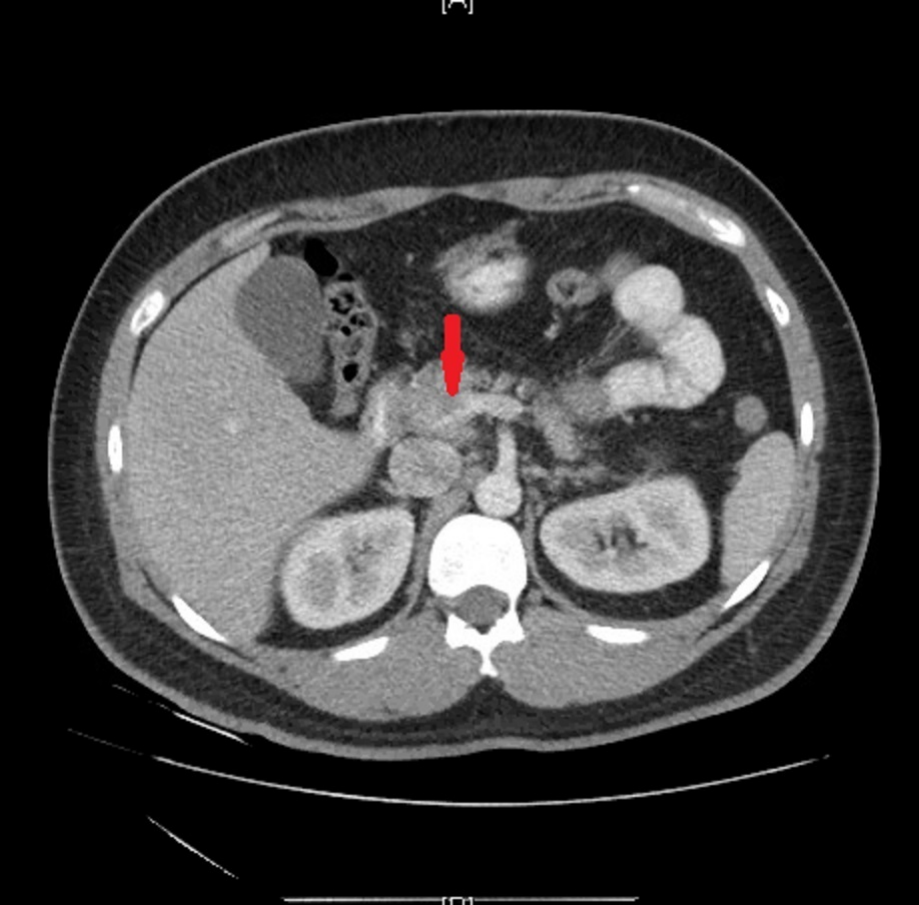
Computed tomography of the abdomen (axial view) showing portal vein thrombus

A 35-year-old male with the history of Klinefelter syndrome on testosterone therapy presented with abdominal pain associated with nausea and fever of two weeks duration. His pain was aggravated by eating and movements. The patient reported that two months previously the dosage of his testosterone shots had been increased from 150 mg intramuscular every two weeks to 200 mg every two weeks to boost his energy levels. On physical exam, his temperature was 101° F; the remainder of his vitals were stable. The abdomen was soft with generalized abdominal tenderness and bowel sounds were normal. Labs, including leukocyte count, hemoglobin, renal function, and hepatic function tests, were normal. Computed tomography (CT) of the abdomen with contrast showed extensive portal vein thrombosis involving the right and left portal veins extending into numerous peripheral branches and heterogeneous liver attenuation secondary to portal vein thrombosis but no focal hepatic lesion was seen (Figure 1). Free testosterone level was 12 pg/ml. Autoimmune panel and thrombophilia workup were negative. The patient was started on full dose enoxaparin, 70 mg every 12 hours (1 mg/kg every 12 hours). A clear liquid diet was started and later advanced as tolerated. Pain control with managed with analgesics. At discharge, the enoxaparin was switched to apixaban, 10 mg twice a day for five days followed by 5 mg twice a day.

## Discussion

KS can cause testicular failure, androgen insufficiency, and impaired spermatogenesis, which results in decreased levels of free testosterone in the serum [3]. KS patients also have an increased risk of autoimmune diseases, endocrinopathies, and thromboembolism [2, 4]. The exact etiology of thrombophilia is unknown. Hypogonadism is associated with hypofibrinolysis and increased synthesis of plasminogen activator inhibitor-1 (PAI-1), which can increase the risk of VTE and cardiovascular diseases [5]. Other factors that may contribute are vascular abnormalities, high factor VIII, factor C and S deficiencies, high homocysteine, antithrombin III deficiency, and factor V Leiden heterozygosity [2].

Supplemental androgens as testosterone replacement therapy are commonly used in the patients with KS. Testosterone replacement therapy (TRT) can inhibit the hypofibrinolysis but they can also be paradoxically prothrombotic which could be dose or duration-dependent [2, 5-6]. Our patient had negative tests for genetic and acquired thrombophilias, so the development of thrombosis was most likely related to the increase in the dose of testosterone replacement therapy since he had been on replacement therapy for over 10 years. The Naranjo drug reaction probability score was 5 and corresponds to the probable relationship between thrombosis and testosterone use [7].

## Conclusions

Klinefelter syndrome increases the risk of thromboembolism. Thrombophilia workup should be done prior to starting testosterone replacement therapy in these patients. Further research is needed to study the role of duration and dose of TRT in development of thrombosis. VTE prophylaxis should be considered in KS patients on TRT. The hypercoagulable state in these cases may need lifelong anticoagulation.

## References

[1] Bojesen A, Juul S, Gravholt CH (2003). Prenatal and postnatal prevalence of Klinefelter syndrome: a national registry study. J Clin Endocrinol Metab.

[2] Glueck CJ, Jetty V, Goldenberg N (2016). Thrombophilia in Klinefelter syndrome with deep venous thrombosis, pulmonary embolism, and mesenteric artery thrombosis on testosterone therapy: A pilot study. Clin Appl Thromb Hemost.

[3] Smyth CM, Bremner WJ (1998). Klinefelter syndrome. Arch Intern Med.

[4] Aoki N (1999). Klinefelter’s syndrome, autoimmunity, and associated endocrinopathies. Intern Med.

[5] Winkler UH (1996). Effects of androgens on haemostasis. Maturitas.

[6] Ozbek M, Öztürk MA, Ureten K (2008). Severe arterial thrombophilia associated with a homozygous MTHFR gene mutation (A1298C) in a young man with Klinefelter syndrome. Clin Appl Thromb Hemost.

[7] Naranjo CA, Busto U, Sellers EM (1981). A method for estimating the probability of adverse drug reactions. Clin Pharmacol Ther.

